# 肺癌围术期患者静脉血栓栓塞症的预防与护理现状调查分析

**DOI:** 10.3779/j.issn.1009-3419.2017.10.01

**Published:** 2017-10-20

**Authors:** 娥 郑, 煜东 唐, 梅 杨, 国卫 车, 嘉妮 张, 娜 杜, 南生 程, 秀英 胡

**Affiliations:** 1 610041 成都，四川大学华西医院胸外科 Department of Thoracic Surgery, West China Hospital, Sichuan University, Chengdu 610041, China; 2 610041 成都，四川大学华西临床医学院 West China School of Medicine, Sichuan University, Chengdu 610041, China; 3 610041 成都，四川大学华西医院 West China Hospital, Sichuan University, Chengdu 610041, China

**Keywords:** 肺肿瘤, 静脉血栓栓塞症, 风险评估, 预防, 护理, Lung neoplasms, Venous thromboembolism, Risk evaluation, Prevention, Nursing

## Abstract

**背景与目的:**

了解我国医院肺癌围术期患者静脉血栓栓塞症（venous thromboembolism, VTE）的预防与护理现状。

**方法:**

采用自行设计的调查问卷，在第一届胸科加速康复外科（enhanced recovery after surgery, ERAS）华西论坛上，对全国108名胸外科护士长进行调查。

**结果:**

① 评估工具与预防规范：97.22%的医院已采用不同评估工具对肺癌围术期患者进行VTE风险分级，其中67.59%的医院已形成VTE预防护理规范。②筛查、预防与随访：56.48%的医院对肺癌患者进行了VTE术前筛查，90.74%的医院对住院患者进行了VTE预防，52.78%的医院对出院患者继续进行了VTE预防，仅有17.59%医院对出院患者VTE的发生情况进行了随访。③不同类型医院肺癌围术期患者VTE预防现状相比，差异无统计学意义（*P* > 0.05），但在VTE风险评估、住院患者VTE预防方面专科医院所有患者已全面实现（100.00%）。

**结论:**

肺癌围术期VTE预防工作已受到广泛重视，但尚缺乏有效的VTE风险评估工具和标准化的VTE预防护理指南。

## Abstract (en)

**Background and objective:**

The purpose of this study was to explore the status of prevention and nursing on venous thromboembolism (VTE) among perioperative patients with lung cancer in Chinese hospital.

**Methods:**

A self-designed questionnaire was used to investigate 108 head nurses from tertiary hospitals during the first West China Forum on Chest Enhanced Recovery After Surgery (ERAS).

**Results:**

(1) Current status of assessment tools and prevention guidelines: 97.22% of the hospitals have carried out VTE risk assessments for surgical patients with lung cancer, 67.59% of the hospitals have established the nursing prevention specifications of VTE. (2) Current status of screening, precaution and follow-up: 56.48% of the hospitals have taken different approach to screen VTE for lung cancer patients in pre-operative period. 90.74% of the hospitals and 52.78% of the hospitals had VTE prophylaxis for hospitalized and discharged patients, but only 17.59% of hospitals were followed up on the incidence of VTE for discharged patients. (3) There was no statistically significant difference in VTE prevention between different type hospitals (*P* > 0.05). But, all patients in the specialist hospital have been fully implemented on VTE risk assessment and VTE prevention (100.00%).

**Conclusion:**

The clinical staff have already realized the importance of VTE prevention, and the VTE prevention in perioperative patients with lung cancer has received extensive attention. But there is still lack of effective risk assessment tools and standardized guidelines of VTE prevention.

静脉血栓栓塞症（venous thromboembolism, VTE）包括深静脉血栓（deep vein thrombosis, DVT）和肺栓塞（pulmonary embolism, PE）两部分，是一种全世界范围内的常见疾病，严重影响患者的生活质量和生命安全。近年来，VTE的发病率呈上升趋势，资料显示，VTE的发病率在美国为（0.96-3.0）/1, 000，欧洲为（0.75-2.69）/1, 000^[1]^。国内虽然没有关于围手术期VTE发病率的确切流行病学报道，但是近年来不同学科各自的报道显示其发病率正逐年攀升^[2-4]^。VTE作为医疗过程中的常见并发症，是患者住院时间明显延长的第二大原因，是心血管意外导致死亡的第三大原因，国际和国内相关指南已将防治VTE列为降低住院患者病死率的最重要策略之一^[5-7]^。肺癌围术期患者并发VTE将延长住院时间，导致高费用、高病死率和高致残率，其预防和治疗应引起临床工作者的充分重视。研究指出，通过护理干预可以有效促进下肢静脉血液回流，预防VTE的发生^[8, 9]^。因此，正确认识VTE，积极采取有效的预防措施，最大程度地保障手术安全和治疗效果，是肺癌围术期护理工作的重要内容之一。本研究旨在了解目前我国肺癌手术患者VTE预防与护理现状，发现VTE预防与护理工作中的不足，为今后完善VTE预防工作提供依据。

## 1 对象与方法

### 1.1 调查对象

采用便利抽样，在第一届胸科加速康复外科（enhanced recovery after surgery, ERAS）华西论坛上，对来自山东、河南、浙江、广东、四川、甘肃、黑龙江、重庆等22个省市的共108位三级医院胸外科护士长进行调查。其中，本科及以上学历者99人，主管护师及以上者87人，护理工作年限10年以上者87人。

### 1.2 调查工具

在广泛查阅文献的基础上，经讨论分析后，形成《肺癌围术期患者静脉血栓栓塞症预防现状调查问卷》，内容包括3个方面：个人信息、医院信息，肺癌围术期患者VTE预防现状。并邀请5名相关专家进行效度检验，结果显示该问卷的CVI为0.91，表明内容效度良好。

### 1.3 资料收集方法

资料收集共分为两个阶段，第一阶段：在第一届胸科ERAS华西论坛上研究者当面向参会胸外科医生和护士长解释本研究的目的与问卷填写方法等，取得知情同意后由其自愿扫描微信二维码填写问卷，当场填写完成后统一提交到管理系统。第二阶段：研究者后台导出数据，采用医生-护士长配对的方式双人核查数据，对于医、护二者填写差异超过5%的数据进行剔除，以确保数据的真实可靠。共计113位护士长参加填写，有效问卷108份，有效回收率为95.58%。

### 1.4 统计学方法

使用SPSS 17.0统计分析软件进行统计学分析。数据的统计描述采用频数与百分比，组间比较采用卡方检验或*Fisher’s*确切概率法，以*P* < 0.05为差异有统计学意义。

## 2 结果

### 2.1 调查对象医院信息

本次共调查108所医院，综合医院居多（82.41%），其中华中地区和西南地区占一半以上（53.70%）。一半左右的胸外科有VTE预防装置，其中58.33%为间歇加压装置（intermittent pneumatic compression, IPC）或足底静脉泵（venous foot pumps, VFP），48.15%为梯度压力弹力袜（graduated compression stockings, GCS），详见表 1。

**1:** 调查对象医院信息（*n*=108） Information of hospitals (*n*=108)

Information		*n*	Percentage (%)
Hospital location	Northeast China	5	4.63
	North China	11	10.19
	East China	9	8.33
	South China	12	11.11
	Central China	29	26.85
	Northwest China	6	5.56
	Southwest China	29	26.85
Hospital type	Specialist hospital	19	17.59
	General hospital	89	82.41
IPC or VFP		63	58.33
GCS		52	48.15
Surgical amount /(times one year)	≤500	48	44.44
	501-1, 000	25	23.15
	1, 001-2, 000	27	25.00
	> 2, 000	8	7.41
IPC: intermittent pneumatic compression; VFP: venous foot pumps; GCS: graduated compression stockings.			

### 2.2 肺癌围术期患者VTE预防现状

97.22%的医院已采用不同评估工具对肺癌围术期患者进行VTE风险分级，其中67.59%的医院已形成VTE预防护理规范。56.48%的医院对肺癌患者进行了VTE术前筛查，分别有90.74%和52.78%的医院采取不同措施对住院患者和出院患者进行了VTE预防，仅有17.59%的医院对出院患者VTE的发生情况进行了随访，详见表 2-表 4。

**2:** 肺癌患者VTE术前筛查现状（*n*=108） Current status of screening on VTE for lung cancer patients (*n*=108)

		*n*	Percentage (%)
Risk assessment tool of VTE		105	97.22
	Caprini	33	30.56
	Khorana	2	1.85
	Padua	1	0.93
	Rogers	4	3.70
	Self-designed questionnaire	62	57.41
	Others	3	2.78
Preoperative screening of VTE		61	56.48
Screening approach	D-Dimer	20	18.52
	D-Dimer and Color Doppler ultrasound of peripheral veins	19	17.59
	D-Dimer and Color Doppler ultrasound of peripheral veins and CTPA	10	9.26
	Color Doppler ultrasound of peripheral veins	7	6.48
	Color Doppler ultrasound of peripheral veins and CTPA	5	4.63
Perioperative precautionary guidelines of VTE		73	67.59
If there will be standardized precautionary guidelines of VTE for thoracic surgery patients, you use it for the possibility	In all probability	63	58.33
	In half probability	32	29.63
	Uncertain	13	12.04
CTPA: CT pulmonary angiography.			

**3:** 肺癌患者围术期VTE预防现状（*n*=108） Current status of prevention on VTE among perioperative for lung cancer patients (*n*=108)

		*n*	Percentage (%)
VTE prevention approach for hospitalized patients		98	90.74
	Mechanical prophylaxis	20	18.52
	Medical prophylaxis	20	18.52
	Mechanical and medical prophylaxis	58	53.70
Onset time of VTE prevention for hospitalized patients	The day before operation	27	25.00
	2-12 hours before operation or 2-12 hours after operation	25	23.15
	The day after operation	41	37.96
	Others	5	4.63
VTE prevention approach for discharged patients		57	52.78
	Mechanical prophylaxis	17	15.74
	Medical prophylaxis	15	13.89
	Mechanical and medical prophylaxis	25	13.15
Time node of VTE prevention for discharged patients	One week after operation	14	12.96
	Two weeks after operation	23	21.30
	Four weeks after operation	15	13.89
	Others	5	4.63

**4:** 肺癌患者VTE术后随访现状（*n*=108） Current status of follow-up on VTE for lung cancer patients (*n*=108)

		*n*	Percentage (%)
Follow-up of VTE for discharged patients		19	17.59
Time node of VTE follow-up	One week after operation	5	4.63
	Two weeks after operation	1	0.93
	Four weeks after operation	8	7.41
	Others	5	4.62
Follow-up approach	D-Dimer	3	2.78
	Color Doppler ultrasound of peripheral veins	6	5.56
	D-Dimer and Color Doppler ultrasound of peripheral veins or CTPA	9	9.25

### 2.3 不同类型医院肺癌围术期患者VTE预防现状

不同类型医院肺癌围术期患者VTE预防比较差异无统计学意义（*P* > 0.05），但在VTE风险评估、住院患者VTE预防方面专科医院所有患者已全面实现（100.00%），详见表 5。

**5:** 不同类型医院肺癌围术期患者VTE预防现状比较[*n* (%)] VTE prevention between different type hospitals [*n* (%)]

	Specialist hospitals	General hospital	*P*
Risk assessment tool of VTE	19 (100.00)	86 (96.63)	0.556
Perioperative precautionary guidelines of VTE	11 (57.89)	62 (69.66)	0.320
Preoperative screening of VTE	11 (57.89)	50 (56.18)	0.891
VTE prevention approach for hospitalized patients	19 (100.00)	79 (88.76)	0.204
VTE prevention approach for discharged patients	9 (47.37)	48 (53.93)	0.603
Follow-up of VTE for discharged patients	4 (21.05)	15 (16.85)	0.440

## 3 讨论

肺癌围术期VTE预防工作已受到广泛重视。证据表明肺癌是常见的合并VTE的肿瘤类型^[10]^，肺癌手术患者麻醉时间超过30 min，发生VTE的风险将增加21倍。一方面由于术中使用镇静剂和肌松剂、组织和血管损伤使机体凝血系统被激活；另一方面术中低血压状态、术后长时间静卧不同程度地使下肢肌肉收缩无力，肌泵作用减低或丧失，引发局部血流滞缓或瘀积，血流变速率处于低剪切状态，血液中被激活的凝血因子和血栓不能及时清除^[3]^，这些均是导致肺癌围手术期患者诱发VTE的高危因素。本研究被调查医院均为三级医院，调查对象的职称、学历水平较高，且有多年临床护理和护理管理工作经验，研究数据的可靠性较高，在一定程度上代表了各省份目前VTE预防现状。研究结果显示，有56.48%的医院已采用不同方式对肺癌患者进行了VTE术前筛查，绝大部分医院（90.74%）根据住院患者的实际情况采取了相应的干预措施，包括机械预防、药物预防或两者相结合的方式，这表明肺癌围手术期患者VTE预防工作已受到医护人员的广泛重视。更加可喜的是，有一半以上的医院对术后出院的患者继续进行了VTE预防，部分医院对出院后患者VTE的发生情况进行了随访与监测，说明目前我国VTE预防工作正逐步由院内向院外发展，这也与国内外肿瘤相关静脉血栓栓塞症预防与治疗专家指南的推荐意见相一致^[11, 12]^。我们可以看到，不同类型的医院肺癌围术期患者VTE预防现状相比较，差异虽无统计学意义（*P* > 0.05），但在VTE风险评估、住院患者VTE预防方面专科医院所有患者均已全面实现（100%）。

缺乏有效的VTE风险评估工具。正确评估和有效识别患者VTE发生的高危因素是预防VTE的基础^[13]^，采用VTE风险评估工具对患者的VTE风险进行分层，针对不同危险层级采取相应的预防措施，不仅能够降低VTE的发生率，减少资源浪费，同时可为建立和健全护理评估与管理程序提供依据^[14]^。ACCP指南也明确指出对非骨科手术患者的VTE预防应根据患者的风险层级进行分级预防^[15]^。目前，我国VTE风险评估尚处于初步探索阶段，尽管医护人员已意识到对患者进行个性化风险评估并进行针对性预防的重要性，但缺乏可靠、有效、实用的风险评估工具^[16]^。由表 2可以看出，参与调查的医院绝大多数（97.22%）已采用相关VTE风险评估量表对肺癌围术期高危患者进行筛查，这一结果虽然远远高于徐园等^[17]^的研究结果，即有31.1%的医院使用患者静脉血栓风险评估表，但不难看出我国目前尚缺乏系统、有效的评估工具。有超过一半的医院（57.41%）为自定评分量表，其敏感性和特异性尚需进一步研究；部分医院为国外引进评估量表，由于不同人群特征和疾病谱，会产生偏倚，且存在人种、体质、文化及生活习惯等方面的差异，其危险因素并不完全适用于我国民众^[18]^，若要在国内推广使用，需结合我国人群疾病特点开展多中心大样本研究。鉴于此，临床VTE预防护理工作需要通过循证依据，找出VTE风险指标、制订风险评估工具，准确评估，根据风险级别分层实施预防护理措施，保证患者安全。

缺少标准化VTE预防护理指南。VTE预防护理指南的制定是VTE预防护理措施落到实处的基础和保障，实施科学、有效的预防措施对于降低VTE的发生、减轻患者痛苦和促进加速康复具有重要意义^[19]^。近年来，我国护理人员在VTE护理预防工作中做了一定探索，形成了一些规范，本研究结果显示有67.59%的医院已形成肺癌手术患者VTE预防规范，且已基本应用于大部分患者；但各医院所采取的规范标准不一、预防水平参差不齐。有研究^[20]^表明，98.7%的护士认为有必要建立静脉血栓防治护理指南。本研究结果也显示如果国内出版标准化胸外科围手术期患者VTE预防指南，87.96%的研究对象表示将采用这一指南。尽管VTE的预防工作已受到各医院的广泛重视，但由于国内尚缺乏相应的护理指南与规范，限制了临床VTE预防护理措施的落实。因此，护理工作者亟需根据我国人群疾病特点与医疗资源现状建立标准化的VTE预防护理指南，使VTE预防工作更加科学、规范，有效推进肺癌围手术期患者的VTE预防。

本研究调查工具为研究者自行设计，调查对象为全国三级医院的护理管理工作者，职称、学历水平较高，样本量相对较小，在人群的选择上存在一定偏倚，研究结果的外推受到一定限制。如有条件今后将会采用随机抽样，扩大样本量，开展多中心研究。
